# Pediatric Urolithiasis: Current Surgical Strategies and Future Perspectives

**DOI:** 10.3389/fped.2022.886425

**Published:** 2022-06-09

**Authors:** Irene Paraboschi, Michele Gnech, Erika Adalgisa De Marco, Dario Guido Minoli, Carolina Bebi, Stefano Paolo Zanetti, Gianantonio Manzoni, Emanuele Montanari, Alfredo Berrettini

**Affiliations:** ^1^Pediatric Urology Unit, Fondazione IRCCS Cà Granda Ospedale Maggiore Policlinico, Milan, Italy; ^2^Department of Urology, Fondazione IRCCS Cà Granda Ospedale Maggiore Policlinico, Università degli Studi di Milano, Milan, Italy

**Keywords:** pediatric urolithiasis, percutaneous nephrolithotomy (PCNL), kidney calculi, children, pediatric stones

## Abstract

New technological innovations and cutting-edge techniques have led to important changes in the surgical management of pediatric urolithiasis. Miniaturized technologies and minimally invasive approaches have been increasingly used in children with urinary stones to minimize surgical complications and improve patient outcomes. Moreover, the new computer technologies of the digital era have been opening new horizons for the preoperative planning and surgical treatment of children with urinary calculi. Three-dimensional modeling reconstructions, virtual, augmented, and mixed reality are rapidly approaching the surgical practice, equipping surgeons with powerful instruments to enhance the real-time intraoperative visualization of normal and pathological structures. The broad range of possibilities offered by these technological innovations in the adult population finds increasing applications in pediatrics, offering a more detailed visualization of small anatomical structures. This review illustrates the most promising techniques and devices to enhance the surgical treatment of pediatric urolithiasis in children, aiming to favor an early adoption and to stimulate more research on this topic.

## Introduction

In the last few decades, there has been a progressive increase in the incidence of pediatric urolithiasis worldwide, which became an important health issue in both low-income developing countries and advanced economies. Several factors have been associated with this increased global incidence, such as genetic, metabolic, anatomical, dietary, infectious, and environmental factors (1).

Predisposing causes for urinary stone disease have been recognized in 75% of children with urinary calculi (2). Urinary stone formation is a complex process, depending on the interaction of different factors, including an increased urinary concentration of stone-forming ions, urinary pH, anatomical factors, that reduce the urinary flow, and metabolic factors, that encourage stone crystallization (3).

In pediatrics, the young age at stone formation requires a precise identification of the underlying cause and personalized treatments to prevent recurrences. Therefore, it is crucial to gather a complete patient medical history, investigate urinary and dietary habits, perform urinary and blood tests, and routinely analyze urinary stone composition. Any risk factors for urinary stone formation should be ruled out and any suspect for upper or lower urinary tract obstruction should be dispelled (3).

Historically, all urinary stones were treated by open surgery. More recently, however, there has been a significant drive toward minimally invasive surgery (MIS), such as extracorporeal shockwave lithotripsy (ESWL), ureteroscopy (URS), retrograde intrarenal surgery (RIRS), and percutaneous nephrolithotomy (PCNL) (4). Beyond the increasing applications of MIS, improvements in miniaturized technologies and fine retrieval instruments and the development of high-power laser disintegration sources are currently revolutionizing the surgical treatment of pediatric urolithiasis. Moreover, new surgical advances and cutting-edge techniques are under development to plan the surgical strategy, increase the stone-free rate e reduce the risk of perioperative complications.

This review article provides an update on the current management of pediatric urolithiasis and illustrates novel technologies and devices to minimize surgical complications and improve patient outcomes.

## Miniaturized Technologies

PCNL is the surgical strategy recommended by international guidelines as the first treatment option for kidney stones larger than 20 mm (5, 6).

In 1976, Fernström and Johansson (7) firstly described this technique, which has significantly evolved in the following decades, becoming a fundamental tool in the armamentarium of pediatric urologists. Standard PCNL access tracts have a size comprised between 24 Fr and 30 Fr. They have the advantages of providing a very high (>90%) stone-free rate in a single session but they are associated with significant treatment morbidity and are not really suitable for children (4).

In this scenario, in 1998, Jackman et al. (8) described, for the first time, a miniaturized surgical technique aiming to decrease the PCNL morbidity in young children. Since then, smaller access sheaths and several miniaturized disintegration technologies have been developed to expand the applications of PCNL in children and decrease the risk of major complications in adults (9).

Due to the increasing use of poorly defined terms, with many studies using overlapping terminology for the same size sheath, in 2016, Wright et al. (9) proposed a standardized nomenclature of the miniaturized PCNL techniques available. Accordingly, to their classification, “mini-PCNL” should be used for access sheaths comprised between 14 Fr and 20 Fr, “ultra-mini-PCNL” between 11 Fr and 13 Fr, and “micro-PCNL” of 4.85 Fr in size (Table 1).

**Table 1:** Summary of the main features of the miniaturized technologies currently available to treat urinary stones in children.

**Terminology**	**Access sheath** **size**	**Nephroscope** **size**	**Reported** **stone-free rate**
Standard-PCNL	22–30 Fr	24 Fr	>90.0% (4)
Mini-PCNL	11–22 Fr	12 Fr	80.6–97.1% (11–13)
Ultra-mini-PCNL	11–13 Fr	6 Fr	95.0% (15)
Micro-PCNL	4.85 Fr	0.9 mm	83.3–86.7% (18, 19)
Mini-micro-PCNL	8 Fr	0.9 mm	NA
Super-mini-PCNL	10–14 Fr	7 Fr	84.7–90.1% (22)

*NA, data not available*.

With regards to the surgical steps, when performing a “mini-PCNL,” the kidney is punctured under ultrasound and radiological guidance. A 16 Fr metallic or self-dilating suctioning access sheath is then placed under fluoroscopic guidance to assist in needle accuracy. Subsequently, a 12 Fr nephroscope is introduced into the collecting system. Stone disintegration is achieved by employing a holmium:YAG laser and stone fragments are then irrigated and suctioned (8–10). In 2020, Baydilli et al. (11) reported their single-institution experience in 206 pediatric patients undergoing mini-PCNL for kidney stones with a success rate of 80.6% after the first session, increasing to 87.9% after auxiliary treatments. In a following study comparing the efficacy and the safety of ultrasonography-guided vs. fluoroscopy-guided “mini-PCNL” in children, Eslahi et al. (12) reported higher stone-free rates: 97.1% in the ultrasonography-guided group (*n* = 35 patients) and 94.3% in the fluoroscopy-guided group (*n* = 35 patients). Similar results were published more recently: Mahmood (13) in 143 patients experienced a 92.4% of stone-free rate after a single “mini-PCNL” session independently by patient's age.

During “ultra-mini-PCNL,” after puncturing the kidney under ultrasound guidance, the dilation is done as a “single-step-dilation” *via* fluoroscopic control. Once the needle is properly positioned into the target calyx, a guidewire is inserted into the collecting system, the needle is removed and a 11–13 Fr working sheath with an obturator is gradually advanced over the guidewire. The obturator is then retracted and a 6 Fr operative mini-nephroscope is inserted into the working sheath. Due to the small size of the instruments adopted, the holmium: YAG laser was used for fragmenting the kidney stones under direct vision. An endoscopic pulsed perfusion system could be employed to inspect the collecting system. Stone fragments and blood clots were expelled by rapidly retracting the endoscope (9, 10, 14). In a recent prospective cohort study comparing “mini-PCNL” vs. “ultra-mini-PCNL” in children, Mishra et al. (15) reported high successful results in both groups (97.5 vs. 95.0%) with no difference in complication rated and decrease in hemoglobin levels.

In “micro-PCNL,” a selective calyceal puncture is made under direct endoscopic vision using a 4.85 Fr “all-seeing needle.” After the retraction of the inner beveled needle, a three-way system is connected to the proximal end of the working sheath. The scope—a fiber the 0.9 mm 10.000 pixel optic—is passed through the connector central port while the other two side ports are used for irrigation and to pass the 200 μm holmium: YAG laser. This technique does not allow any fragment retrieval and represents a precise under vision lithotripsy, stone clearance relies mainly on spontaneous passage of fragments and powder obtained by lithotripsy and on cautious pressurized irrigation (9, 10, 16, 17). In a retrospective study including 24 infants undergoing “micro-PNL” for renal stones, Dede et al. (18) recorded a stone-free rate of 83.3% with 4 patients complaining of post-operative renal colic and 2 patients experiencing post-operative fever. Similarly, a stone-free rate of 86.7% was reported in a recent paper involving 15 patients undergoing “micro-PCNL” for kidney stones located in lower calyx (19).

“Mini-micro-PCNL” is a technical variation of “micro-PCNL.” It employs a 8 Fr metallic sheath attached to the same three-way system of standard “micro-PCNL” to prevent its propensity to bend during manipulation and stone treatment. This modification theoretically allows an easier intrarenal manipulation from one calyx to another whilst allowing the insertion of a 1.6 mm ultrasonic lithotripter to aid stone fragmentation and suction (9, 20).

“Super-mini-PCNL” is a new device developed to overcome some of the limitations owing to the miniaturized PCNL techniques. These include a limited continuous irrigation flow, a poor endoscopic visualization, a difficulty in stone fragment extraction, and the risks connected to elevated renal pelvic pressure (21).

The “Super-mini-PCNL” is mainly composed of a 7 Fr nephroscope with increased irrigation function and a modified nephrostomy access sheath with suction-evacuation capability (21). One hundred and eleven children with kidney stones treated with the “Super-mini-PCNL” were reviewed in a retrospective study published by Liu et al. (22). The rate of complete stone clearance was 84.7% on post-operative day 1 and 90.1% at the 3-month follow-up. Seventeen (15.3%) children developed complications: 10 were scored grade I and 7 grade II, according to the Clavien-Dindo classification system. In a following study (23), the safety and efficacy of the “Super-mini-PCNL” technique for pediatric kidney stones <25 mm (*n* = 111) was compared with the standard ESWL procedure (*n* = 108). The prevalence of residual fragments after ESWL was significantly higher when compared with “Super-mini-PCNL.” While the ESWL procedure required multiple sessions under general anesthesia in 54.6% of cases, the “Super-mini-PCNL” was successful in just one session in all cases. No major complications occurred, and no blood transfusions were necessary while minor complications were observed with similar rates in both groups of patients.

## Semi-Closed-Circuit Vacuum-Assisted Technologies

**Figure 1:**
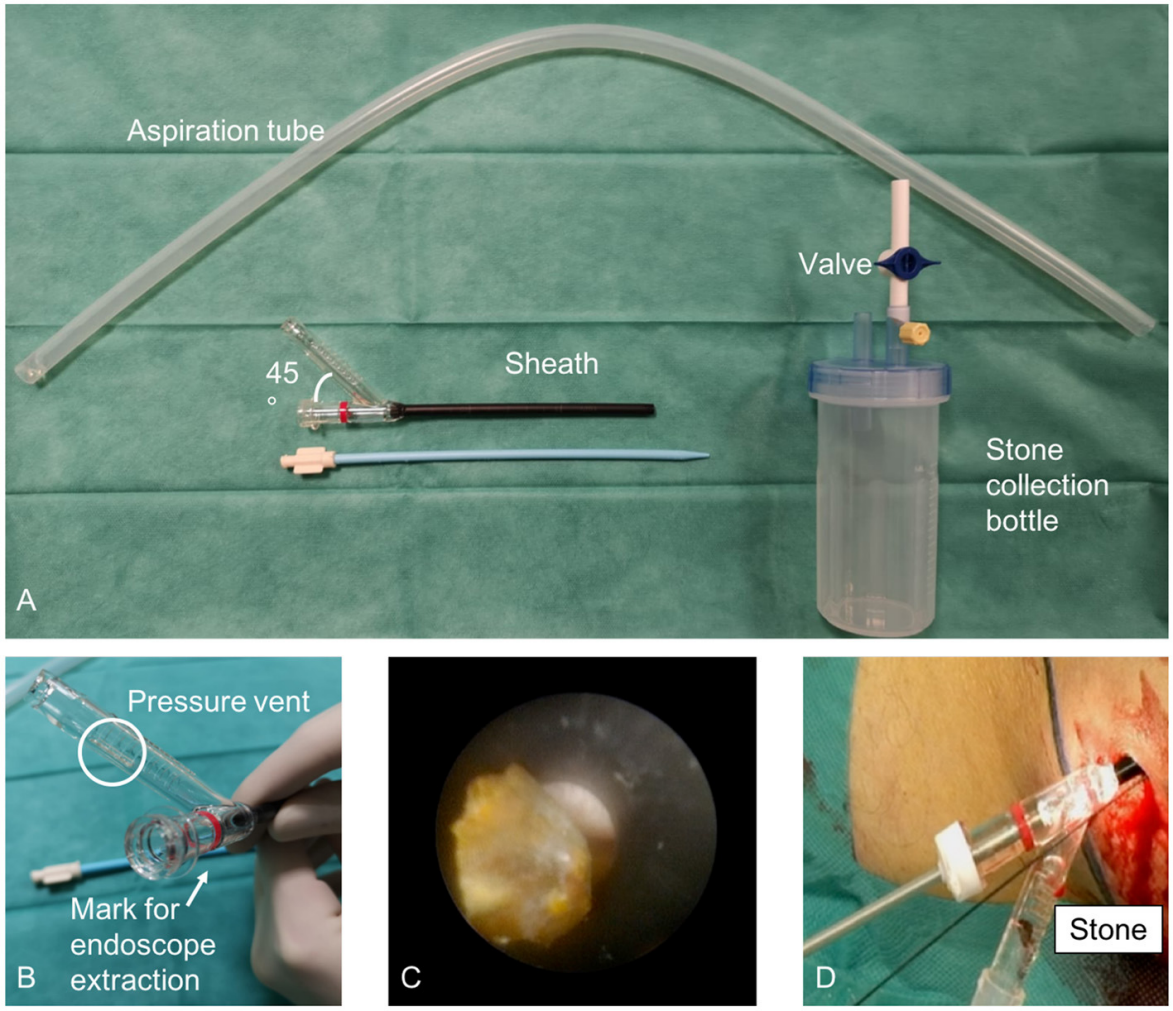
Semi-closed-circuit vacuum-assisted mini-PCNL (vmPCNL) performed by using the ClearPetra^®^ access sheath (Well Lead Medica Co. Ltd., China). Courtesy of Gallioli et al. (25).

While the miniaturization of PCNL technologies have reduced the risks of major surgical complications, some authors argued that it could hinder surgical and functional outcomes. To overcome the limits of standard PCNL, a semi-closed-circuit vacuum-assisted mini-PCNL (vmPCNL) system has been developed (24, 25). In 2020, Gallioli et al. (25) published the results of 18 consecutive vmPCNLs performed in 13 children by using the ClearPetra^®^ access sheath (Well Lead Medica Co. Ltd., China) equipped with a lateral arm connected to an aspiration system by a plastic stone connector (Figure 1). They reported a stone-free rate of 81.3% in 18 procedures lasting 128 min (IQR: 99–167). Neither intraoperative complications occurred nor blood transfusions were required. More recently, the safety and feasibility of vmPCNL for kidney stone treatment in the pediatric population was proved in a video article (24). Twelve vmPCNLs were performed in 8 patients using the 16 Fr ClearPetra^®^ nephrostomy sheath (Well Lead Medica Co. Ltd., China). The continuous inflow and the suction-controlled outflow ensured a clear vision during lithotripsy while maintaining a low intrarenal pressure. All these factors seemed to reduce the operative time (median: 108 min; range: 60–184 min) and achieve a satisfactory stone-free rate (80.0%). To go further, in 2021, Quiroz et al. (26) published the video of a 15 month-old boy with a staghorn calculus undergoing an “ultra-mini PCNL” assisted by using the ClearPetra^®^ suction-evacuation access sheath and a warming irrigation fluid system (Rocamed^®^). The warming irrigation fluids offered the advantages of preventing heat loss and the possible hypothermia associated with the use of anesthetics, the prolonged skin exposure, and the administration of large volumes of intravenous and irrigation fluids.

## Robotic-Assisted Laparoscopic Procedures

Although the majority of children with urinary stones are managed *via* ESWL, URS, RIRS, or PCNL, the use of MIS has significantly increased over the last two decades (27, 28).

The increasing use of dedicated pediatric instrumentation has significantly reduced the risks of complications while the advent of the robotic platform has expanded the use of MIS in children.

Accordingly to the European Association of Urology (EAU) guidelines for the management of urinary stone disease in children (29) minimally invasive approaches are good alternatives in patients with a history of previous failed endoscopic procedures, complex renal anatomy (ectopic or retrorenal colon), concomitant ureteropelvic junction obstruction (UPJO) or caliceal diverticula, mega-ureter, or large impacted stones. In a multicentric international study published in 2021, Esposito et al. (28) reported the feasibility and safety of the robotic-assisted laparoscopic surgery for the surgical treatment of 15 children with complex urinary stones: 11 patients underwent a simultaneous robotic-assisted pyelolithotomy and pyeloplasty for concomitant UPJO, 2 patients received a robotic-assisted pyelolithotomy for isolated staghorn stones and 2 patients required a robotic-assisted cystolithotomy for bladder stones.

Roth et al. (30) published the largest series of 26 children with complex nephrolithiasis inaccessible by standard treatments who underwent an endoscopic-assisted robotic pyelolithotomy. By using a flexible endoscope passed through a robotic trocar, the renal collecting system was explored and the urinary stones were treated *via* pyelolotomy or, when necessary, fragmented with laser lithotripsy. The authors concluded that this technique was an effective management option for stone treatment, with a stone-free rate of 70.4% following a primary procedure and 96.3% following a secondary procedure, with less than one-third of patients having any residual stone burden following the initial surgery and requiring a secondary procedure.

## Three-Dimensional Reconstruction Technologies

Despite the introduction of miniaturized instruments in PCNL procedures, the percutaneous kidney puncture still represents the most challenging step. This procedure is characterized by the steepest learning curve due to the risk of damaging the surrounding blood vessels and organs. This is even more true in the case of staghorn stones, kidney stones associated with renal calyx neck stenosis or dilation, or when an abnormal kidney anatomy is present. Therefore, intraoperative ultrasound scans and X-rays are required to establish the most appropriate working route to reach the target renal calyx and avoid damaging the surrounding structures. However, these radiological techniques are two-dimensional imaging modalities with low-resolution and limited usefulness when performing complex surgeries. Hence, more advanced imaging technologies have been developed to assist surgeons in establishing a safe and reliable percutaneous renal access.

In particular, three-dimensional computed tomography (3D-CT) reconstructions have been adopted to facilitate comprehensive planning for PCNL (31, 32). In 2021, Tan et al. (32) published a retrospective comparative study including 139 patients with complex renal calculi undergoing PCNL. In 72 cases the procedure was preceded by 3D-CT reconstruction techniques. Worth noting, in this group of patients, the operation time and the incidence of post-operative complications were significantly lower while the initial stone clearance rate and the first-time puncture success rate were significantly higher.

Not only 3D-CT reconstructions but also 3D printing technologies have been used to plan PCNL in case of complex renal calculi. In 2022, Cui et al. (33) run a randomized controlled trial to investigate the efficacy and the safety of 3D printing combined with PCNL for the treatment of kidney stones. The 3D printed model of the urinary tract was used to communicate with patients and help them to fully understand the indications for surgery and the surgical approach needed, the expected results, the surgical risks, and the possible post-operative complications. Moreover, by using the imaging data and 3D printing model, surgeons were able to properly plan the PCNL access, the target renal calyx, the puncture angle, the predicted residual stone, the puncture depth, and the lithotripsy process. The result of the study showed that the operation time and the hospital stay were shorter, the blood loss and the incidence of perioperative complications were lower, the stone-free rate was higher while the patient-doctor communication was more effective in the group of patients undergoing 3D printing combined with PCNL compared to the control group. The authors concluded that 3D printing was a safe and effective method to assist surgeons in planning PCNL for patients with complex kidney stones.

## Virtual Reality

Virtual reality is an interface generated by a computer using multi-sensor technologies that shows an interactive and simulated environment to the user. Thanks to the visual input coming from the head-mounted display, the external world is replaced by one developed by a computer, allowing the users to dive into a novel virtual world. VR is increasingly used in medicine for many purposes, in particular in surgical training, education, and preoperative planning (34, 35).

In this scenario, VR has also been adopted to create patient-specific 3D kidney models that have been used to obtain a detailed definition of kidney stones and their relationship with the surrounding anatomical structures. In 2019, Parkhomenko et al. (36) run the first pilot study evaluating the efficacy of VR technology for planning 25 PCNL. The authors developed a patient-specific CT-based VR model that immersed the observer in an interactive 3D reality, simulating the patient's renal anatomy. Using VR, urologists were able to visualize and manipulate all the relevant anatomic structures including the renal parenchyma, the collecting system, the kidney stone, and the surrounding organs and blood vessels. The results of the study showed that the VR model improved surgeons' understanding of the individual patient's anatomy and helped them to plan the most appropriate working route to reach the target renal calyx during PCNL, consequently altering the surgical approach in 40% of cases. Moreover, when compared to 25 retrospectives matched-paired PCNL performed without the VR model, it significantly reduced the blood loss and the fluoroscopy time, decreased the number of nephrostomy tracts, and increased the stone-free rate. In addition, using VR, patients improved their understanding of the planned surgical procedure, which helped them to deal with anxiety.

## Augmented Reality

Augmented reality (AR) is the superimposition of computer-generated images over a user's view of the real world. In AR, multimedia elements (such as computer-generated images, audio, and video) are added to the real-world environment to enhance its experience. Unlike VR applications, in AR, the real-world environment is presented to the user but enriched and modified with computer-assisted additions. AR has a broad area of applications in medicine, especially in the field of urology (34).

Since one of the most challenging maneuvers in PCNL is the correct access to the collecting system, AR technologies, mathematical calculation software and 3D modeling have been exploited for calculating the correct access point and angle for PCNL. In this regard, Rassweiler et al. (37) reported their clinical experience with an iPad^®^-assisted marker-based navigation for the percutaneous access to the kidney during PCNL. Initially, a picture of the patient was taken using the iPad^®^ as a camera. Subsequently, an algorithm was generated by a server linked to the iPad^®^ to identify the position and the orientation of the navigation system and to overlap it with the preoperative images made using a segmented CT scan. The proper superimposition of the virtual markers onto the real world provided a virtual insight into the patient. In a following study, Müller et al. (38) compared the puncturing time and the radiation exposure of a kidney phantom during 53 kidney punctures performed by a urological trainee and two experts, using an iPad^®^ navigation system, ultrasound and fluoroscopy. With regards to the puncturing time, the trainee outperformed with the proposed AR system whereas the experts did best with fluoroscopy. In terms of radiation exposure, the iPad^®^ assistance significantly lowered it for both the trainee and the experts.

## Mixed Reality

Mixed reality (MR) technology combines virtual and real-world images into a new real environment, by combining VR and AR technologies. The most significant innovation that characterizes this new tool is that the computer-generated virtual images and data can interact with and used by users in real time (34).

In this regard, Porpiglia et al. (39) recently developed 3D MR holograms of the kidney anatomy that were overlapped onto the surgical field to establish the access point and guide the needle puncture during 10 endoscopic combined intrarenal surgeries (ECIRS) for kidney stones. A comparative analysis with a retrospective series of patients who underwent the standard procedure showed a significantly shorter radiation exposure time and a higher success rate for kidney puncture at the first attempt for the 3D MR holograms group.

## Near-Infrared Fluorescent Probes

Current methods for the preoperative assessment of urolithiasis include plain radiograph, fluoroscopy, ultrasonography, and non-contrast CT scan. Conversely, the intraoperative visualization of urinary calculi primarily depends on fluoroscopy and ultrasonography. These imaging modalities are burdened by low sensitivity and specificity when treating urinary stones, mainly due to the small size of the urinary stones/fragments and their hidden location (40). Therefore, alternative imaging techniques are urgently needed for the surgical treatment of stone disease.

The fluorescence signal in the first near-infrared window (NIR-I, 700–900 nm) has increased imaging depth compared to conventional imaging in the visible region (400–700 nm) and has demonstrated great potential in both biomedical research and clinical practice (41–43).

In 2008, Figueiredo et al. (40) illustrated a new method for the intraoperative detection of calcium urolithiasis using two commercially available far-red (OS680) and NIR-I (OS750) fluorescent probes. Once administered, these diphosphonate imaging agents bound to hydroxyapatite, the major mineral product of osteoblasts and calcifying vascular cells, and, thus, rendered fluorescent various calcium calculi with mixed composition.

Compared to the NIR-I, the fluorescence signal in the second near-infrared window (NIR-II, 1,000–1,700 nm) offers higher penetration depth and spatial resolution with improved signal-to-background ratios because of low photon scattering and minimal tissue autofluorescence (44, 45). In this regard, by injecting NIR-II 2TT-oC6B dots into the ureter and using an indium gallium arsenide (InGaAs) camera, a filling defect was clearly seen in an animal model of ureteral stone, indicating the precise location of the urinary stone (44, 45).

NIR-I probes have been exploited in preclinical studies not only to identify renal (40) and ureteral (44, 45) stones but also to guide complex MIS (46). In this regard, in a recent paper published by Sood et al. (46), the administration of the indocyanine green (ICG) NIR-I fluorescent dye helped to guide the renal parenchyma incision in an avascular plane during 2 robotic anatrophic nephrolithotomies in a porcine model of staghorn stones.

## Conclusions

Miniaturized instruments and new technological developments have led to many innovations in the surgical management of pediatric urolithiasis. The devices and technologies described in this review article have the potential to revolutionize the surgical planning and treatment of urinary stones in children. Pediatric urologists need to keep up to date with these recent technological advances that will possibly reach the clinical practice soon.

## Author Contributions

AB and IP: study concept and design. IP, MG, DM, ED, CB, and SZ: acquisition of data. AB, IP, and EM: analysis and interpretation of data. AB, EM, and GM: supervision. All authors contributed to the article and approved the submitted version.

## Conflict of Interest

The authors declare that the research was conducted in the absence of any commercial or financial relationships that could be construed as a potential conflict of interest.

## Publisher's Note

All claims expressed in this article are solely those of the authors and do not necessarily represent those of their affiliated organizations, or those of the publisher, the editors and the reviewers. Any product that may be evaluated in this article, or claim that may be made by its manufacturer, is not guaranteed or endorsed by the publisher.
